# Revisiting the Affect Regulation Model of Suicide Ideation: Bidirectional Effects of Momentary Suicide Ideation and Affect

**DOI:** 10.1111/sltb.70027

**Published:** 2025-05-22

**Authors:** Lori N. Scott, Sarah L. Brown

**Affiliations:** ^1^ Department of Psychiatry University of Pittsburgh School of Medicine Pittsburgh Pennsylvania USA; ^2^ Department of Psychology Florida State University Tallahassee, Florida USA

**Keywords:** affect regulation, dynamic structural equation modeling, ecological momentary assessment, negative affect, positive affect, suicide ideation

## Abstract

**Introduction:**

Suicide ideation (SI) may provide relief from negative affect (NA), thereby making future SI more likely through reinforcement processes. Though some studies suggest that SI reduces NA, this evidence is limited and inconclusive.

**Methods:**

We used dynamic structural equation models to test whether bidirectional associations between SI and both NA and positive affect (PA) over 2‐h intervals support an affect regulation model of SI. Participants were 140 young adults with SI or suicidal behaviors in the past 4 months who completed a 21‐day ecological momentary assessment with seven daily assessments.

**Results:**

Results demonstrated reciprocal effects over 2‐h intervals, such that within‐person increases in NA and decreases in PA were associated with subsequent increases in SI, and increases in SI were associated with subsequent increases in NA and decreases in PA. The occurrence of SI did not significantly moderate the persistence of NA or PA over 2‐h intervals. However, we replicated prior findings of decreases in affective distress following SI when analysis was restricted to occasions when SI occurred and resolved by the next occasion.

**Conclusions:**

Results suggest that distinct analytic and sampling methods can lead to divergent conclusions regarding whether SI provides relief from affective distress.

## 1 Introduction

Suicide ideation (SI), defined as thinking about, considering, or planning suicide, is one of the strongest known predictors of suicide attempts (Franklin et al. 2017; Nock et al. 2018). In annual surveys from 2008 to 2019, 4%–4.8% of adults in the United States reported having serious thoughts of suicide in the past year (Substance Abuse and Mental Health Services Administration 2020). SI fluctuates within individuals over time (Kleiman et al. 2017) and tends to persist or reoccur over up to 1 year of follow‐up (Nock et al. 2008; Suárez‐Pinilla et al. 2020). Given the high prevalence of SI, its dynamic and persistent nature, and its potency as a risk factor for suicidal behavior and death by suicide, it is critical to understand factors that predict the maintenance and exacerbation of SI over varying time scales in at‐risk populations.

People often report thinking of suicide to regulate or escape from painful emotions, and the use of SI to regulate affect is associated with increased frequency and severity of SI over time (Coppersmith et al. 2023). The “affect regulation” (aka, “reinforcement”) model of SI (Kleiman et al. 2018; Kuehn, Dora, et al. 2022) proposes that SI functions as a form of emotion regulation or escape from negative affect (NA), which may be reinforcing and contribute to the maintenance of SI. In other words, to the extent that increases in SI are closely followed by subsequent decreases in NA, or even by increases in positive affects (PA) such as calm or relaxation, SI may be reinforced by relief from aversive emotional states, making future SI more likely when affective distress recurs. Thus, an affect regulation model of SI has two components: (1) SI is preceded by increases in NA and/or decreases in PA and (2) SI is followed by decreases in NA and/or increases in PA. This theory has high clinical relevance, as it might explain why SI often persists within individuals over time and point to clear clinical targets for intervention.

Ecological momentary assessment (EMA) is ideal for testing affect regulation models of SI because it provides the opportunity to examine how SI and affective experiences dynamically influence one another in real life. Numerous EMA studies have found that acute increases in SI tend to be preceded by increases in NA (e.g., Kuehn, Dora, et al. 2022; Kuehn, Foster, et al. 2022; Nock et al. 2009; Victor et al. 2019) and/or decreases in PA (Husky et al. 2017; Kaurin et al. 2022a). Thus, the first component of the affect regulation model (i.e., SI is preceded by increased NA and/or decreased PA) is well established.

However, the second component of the affect regulation model (i.e., SI is followed by decreased NA and/or increased PA) has received mixed support. Kleiman et al. (2018) examined change in NA and PA from before to after SI in pairs of consecutive EMA assessments within 8 h of each other where any SI was present followed by an absence of SI at the next timepoint. They found that NA decreased and PA increased following SI, consistent with the second component of the affect regulation model. Using the same analytic method, Coppersmith et al. (2023) also found that NA decreased following SI with pairs of EMA assessments up to 4 h apart. Still using the same method, Kuehn, Dora, et al. (2022) were able to replicate Kleiman et al.'s (2018) findings regarding decreases in NA after SI in raw data from 13 intensive longitudinal datasets. However, all these analyses were limited in that they used pairs of consecutive assessments where there was SI followed by no SI at the next occasion. This approach may bias results because sequences of continued SI across occasions (i.e., when SI may not be effectively regulating affect) are excluded. In addition, this approach does not account for temporal bidirectional effects or autoregressive effects of SI and affect over time. Modeling autoregressive effects helps to avoid erroneously attributing variance in affect outcomes to SI, and vice versa, with a critical implication for an interpretation focused on change. To address these issues, Coppersmith et al. (2023) conducted an additional analysis using all available data to examine the bidirectional associations between SI and NA over 4‐h intervals. They found positive reciprocal effects between SI and NA (i.e., SI predicted subsequent increases in NA 4 h later), which runs counter to hypotheses implied by an affect regulation model. These results suggest that different methodological approaches to examining the same datasets may lead to opposite conclusions, lending mixed support for an affect regulation model and suggesting more complexity in associations between affective experiences and SI to be explored.

Other recent EMA studies have shown further evidence for increases in NA or inconsistent decreases across NA states following SI. For example, Al‐Dajani and Uliaszek (2021) found greater NA intensity across NA categories (e.g., anger, anxiety) following SI onset when participants were asked to retrospectively reflect on emotions before and after SI. However, when they examined pairs of consecutive emotional intensity assessments before and after occurrences of SI (i.e., similar to previous studies described above), they found reductions in stress and anxiety (but not other NA categories) following SI. Rath et al. (2019) found evidence of increased distress and reduced PA immediately following the occurrence of SI in a network analysis of EMA data, which models all available EMA responses as a network of interconnected nodes and edges, revealing patterns of associations and structural dependencies. Taken together, results from these studies suggest that SI may be followed by an immediate increase in affective states that may be distressing (i.e., increased NA and/or decreased PA), although Al‐Dajani and Uliaszek's (2021) findings suggest that perhaps only specific NA states (i.e., anxiety and stress) may dissipate over subsequent hours.

To summarize, results from EMA studies examining shifts in affect following SI are inconclusive and yield mixed support for the second, and arguably the most critical, component of the affect regulation model of SI (i.e., whether SI is followed by reduced NA and/or increased PA, which would suggest that SI regulates affect in a way that could be reinforcing). Most prior studies that have directly tested this model have not addressed issues of temporal directionality or autoregressive effects, and almost all have focused on selected pairs of responses before and after times when SI occurred (and only when SI resolved at the next occasion) rather than utilizing all available data. Only including consecutive pairs of responses where SI resolves inherently restricts analysis to times when SI is potentially reinforcing. Given these limitations and inconsistencies in the literature, the question of whether SI provides relief from aversive affective states is far from resolved.

To address these limitations and inconsistencies, we examined whether the bidirectional effects of changes in SI and both NA and PA over short time intervals are consistent with an affect regulation model of SI using EMA data from young adults with recent SI or suicidal behavior. Based on recent findings (Coppersmith et al. 2023), we hypothesized reciprocal associations such that SI would be preceded and followed by increased NA and decreased PA, which would not fully support the critical second component of the affect regulation model. We also conducted exploratory moderation analyses to examine whether experiencing SI moderated the persistence of NA or PA (i.e., inertia) over 2‐h time intervals. Evidence consistent with an affect regulation model would show that SI dampens the persistence of NA or strengthens the persistence of PA over 2‐h intervals. Given the novelty of this approach to examining this question, and evidence that different analytic approaches lead to distinct conclusions, we did not make specific hypotheses for the moderation analyses. Finally, to help further clarify the impact of methodological choices, we conducted additional analyses to attempt replication of findings from previous studies that used only selected pairs of responses and did not account for autoregressive or reciprocal effects.

## 2 Methods

### 2.1 Participants

One‐hundred and fifty participants were recruited from July 2019 through April 2023 for the Daily Emotions and Relationships (DEAR) study, which is a longitudinal study of suicide risk in a transdiagnostic sample of young adults. Individuals with recent SI or suicidal behavior were identified using advertisements in community outpatient clinical settings and an online research registry. To be eligible, participants were 18–35 years old, had SI or suicidal behaviors within the past 4 months per the Columbia Suicide Severity Rating Scale (C‐SSRS; Posner et al. 2011), and were receiving ongoing behavioral healthcare. Of the 150 enrolled, 141 participants began the EMA protocol. One participant who only completed two EMA surveys was excluded. Thus, 140 participants were included. See Table 1 for demographics information.

**TABLE 1:** Demographic information (*N* = 140).

	*n* (%)
Sex assigned at birth	
Male	24 (17.1)
Female	116 (82.9)
Gender	
Male	21 (15.0)
Female	103 (73.6)
Transgender male‐to‐female	2 (1.4)
Transgender female‐to‐male	4 (2.9)
Do not identify as male or female	9 (6.4)
Other	1 (0.7)
Ethnicity	
Non‐Hispanic/LatinX	127 (90.7)
Hispanic/LatinX	13 (9.3)
Race	
Asian	11 (7.9)
Black or African American	22 (15.7)
White	96 (68.6)
More than one race	10 (7.1)
Prefer not to respond	1 (0.7)
Sexual orientation	
Heterosexual/straight	67 (47.9)
Gay/lesbian/homosexual	14 (10.0)
Bisexual	37 (26.4)
Other	16 (11.4)
Not sure	4 (2.9)
Declined	1 (0.7)
Education	
Less than high school or GED	1 (0.7)
High school or GED	11 (7.9)
Some college	47 (33.6)
Technical/trade school certificate	4 (2.9)
College degree or higher	77 (55.0)
Employment	
Not employed	39 (27.9)
Part‐time	46 (32.9)
Full‐time	55 (39.3)
Annual income: *M* (SD)	$73,546 ($77,451)
Public assistance (e.g., Medicaid, WIC) in last year	49 (35.0)

### 2.2 Procedures

Participants completed diagnostic and suicide history interviews followed by a 21‐day web‐based EMA protocol using smartphones. All participants provided written and oral informed consent, and study procedures were conducted in accordance with the responsible Institutional Review Board.

### 2.3 Interviews

Psychiatric disorders, SI, and suicide behavior histories were assessed using the Structured Clinical Interview for DSM‐5 Research Version (SCID‐5‐RV; First et al. 2015), selected items from the Structured Clinical Interview for DSM‐IV Personality Disorders (SIDP‐IV; Pfohl et al. 1997), and the C‐SSRS (Posner et al. 2011). Interviewers were clinicians at the postbaccalaureate, master's, and doctoral levels who were trained by licensed doctoral‐level clinical psychologists. Clinical and diagnostic information are presented in Table 2.

**TABLE 2:** Clinical and diagnostic information (*N* = 140).

	*n* (%)
C‐SSRS SI highest level (past 4 months)	
Wish to be dead	23 (16.4)
Nonspecific active SI	24 (17.1)
Active SI with methods (no plan or intent)	41 (29.3)
Active SI with some intent (no plan)	44 (31.4)
Active SI with specific plan and intent	8 (5.7)
C‐SSRS suicide behavior (past 4 months)	
Actual attempt	7 (5.0)
Interrupted attempt	4 (2.9)
Aborted attempt	7 (5.0)
Preparatory behavior	14 (10.0)
C‐SSRS suicide behavior (lifetime)	
Actual attempt	52 (37.1)
Interrupted attempt	17 (12.1)
Aborted attempt	40 (28.6)
Preparatory behavior	55 (39.3)
SCID‐5‐RV current diagnosis	
Mood disorder	89 (63.6)
Anxiety disorder	91 (65.0)
Obsessive‐compulsive disorder	17 (12.1)
Trauma and stress‐related disorder	31 (22.1)
Substance use disorder	39 (27.9)
Eating disorder	8 (5.7)
SCID‐5‐RV lifetime diagnosis	
Mood disorder	139 (99.3)
Anxiety disorder	110 (78.6)
Obsessive‐compulsive disorder	29 (20.7)
Trauma and stress‐related disorder	65 (46.4)
Substance use disorder	75 (53.6)
Eating disorder	43 (30.7)
SIDP‐IV current diagnoses	
Borderline personality disorder	20 (14.3)
Antisocial personality disorder	5 (3.6)
Avoidant personality disorder	35 (25.0)

*Note: N* = 140. C‐SSRS = Columbia Suicide Severity Rating Scale (Posner et al. 2011); SCID‐5‐RV = Structured Clinical Interview for DSM‐5 (First et al. 2015); SIDP‐IV = Structured Clinical Interview for DSM‐IV Personality Disorders (Pfohl et al. 1997), the criteria for which are unchanged in DSM‐5. Percentages may not sum to 100% due to missing data.

### 2.4 EMA Protocol

Seven semi‐random surveys per day were scheduled between participants' self‐reported typical wake and sleep times for a maximum of 147 possible prompts over 21 days (for details, see Cox et al. 2023 and https://osf.io/7c4yq/). Mean compliance (proportion of possible surveys completed) was 87%. A total of 17,455 observations were available for this analysis. The median time interval between assessments was 2.20 h.

### 2.5 Measures

#### 2.5.1 Suicide Ideation

##### 2.5.1.1 Dimensional SI

At each EMA prompt, participants rated the following statements intended to capture both passive and active aspects of SI on a scale of 0 (*Not strong at all*) to 100 (*Very strong*): “At this moment, how strong is your…wish to live (reverse‐scored), wish to die, desire to die by suicide.” These selected items were based on items from the Beck Suicide Scale (Beck and Steer 1991) and have been used in previous EMA studies (e.g., Coppersmith et al. 2019), typically using a 3‐point scale. We administered these items on a 0–100 scale in order to maintain consistency in how affect items were scaled within the EMA surveys and to maximize variability in response options (i.e., to capture SI along a dimensional continuum). To make the scale comparable across occasions even if there are occasional missing data points for individual items, these items were averaged to create a momentary dimensional SI score. Coefficient omega (*ω*), calculated using multilevel confirmatory factor analysis in Mplus 8.10 (Muthén and Muthén 2023) according to McDonald's (1999) formula (see also Shrout and Lane 2012; Bolger and Laurenceau 2013), demonstrated satisfactory within‐person internal consistency for this scale (*ω* = 0.73). The intraclass correlation coefficient (ICC) for the SI scale was 0.69, indicating that 31% of variability in dimensional SI was within individuals. Scores were moderately skewed (skewness = 1.28) but fell within recommended levels suitable for robust estimation methods used here (e.g., see Kline 2011).

##### 2.5.1.2 Binary SI

Participants were asked at each prompt to report whether they had experienced active SI since the last completed assessment using an item adapted for self‐report from the C‐SSRS (Posner et al. 2011), “Since my last completed survey [date/time], I thought about killing myself” (yes/no). Binary SI was endorsed at least one time during EMA by 81 participants (57.9%) and on 3.5% of all occasions. The ICC for binary SI was 0.54, indicating that 46% of the variance was within persons over time. The average within‐person correlation between dimensional and binary SI endorsement was *r* = 0.46 (*p* < 0.001), suggesting that these items assessed related yet distinct constructs.

#### 2.5.2 Positive and Negative Affect

At each EMA prompt, participants rated the degree to which they were experiencing several mood states in randomized order using the statement “At this moment I feel… (mood)” on a scale of 0 (*Not at all*) to 100 (*Very much*). Mood items were selected to represent high‐arousal and low‐arousal positive and negative affect based on the circumplex model of affect (Russell 1980) and consistent with prior EMA work (e.g., Rowland et al. 2020). PA scores were comprised of the mean of momentary ratings for happy, excited, relaxed, and satisfied (*ω* = 0.80; ICC = 0.45; skewness = 0.21). NA scores were comprised of ratings for sad, depressed, anxious, angry, tired, and ashamed (*ω* = 0.74; ICC = 0.62; skewness = 0.53).

### 2.6 Data Analytic Strategy

Hypotheses were tested using two‐level dynamic structural equation models (DSEM) using Bayesian Markov Chain Monte Carlo (MCMC) estimation with default (i.e., noninformative) priors in Mplus 8.10 (Muthén and Muthén 2023). Noninformative priors let the data drive the estimation rather than introducing prior assumptions, which reduces unintended bias that can result from introducing priors when there is limited past research from which to draw informative priors (Asparouhov et al. 2018). Bayesian MCMC estimation is robust for complex non‐normally distributed data (Muthén 2010) and uses all available data without dropping cases or excluding participants with partially missing data (for details, see Asparouhov et al. 2018). Repeated PA, NA, and SI measures were automatically decomposed into within‐ and between‐person variance using latent mean centering. To account for unequal intervals between observations, we specified the time interval for time‐lagged associations using TINTERVAL = 2 h based on the median time between assessments of 2.2 h. We included time elapsed since the first EMA assessment (“days,” person‐mean‐centered) as a within‐person predictor in all models, which accounts for trends over time in outcomes (e.g., decreases in NA, PA, or SI over the course of the study). We allowed all random effects to covary with each other at the between‐persons level. Models were estimated with 50,000 iterations and two MCMC chains. Model convergence was checked using the proportional scale reduction (PSR) indices for each parameter and examining trace plots for irregularities (e.g., trends and cycles). PSR values close to 1 suggest model convergence. Final PSR values for the DSEM models ranged from 1.001 to 1.063, indicating good convergence.

**FIGURE 1:**
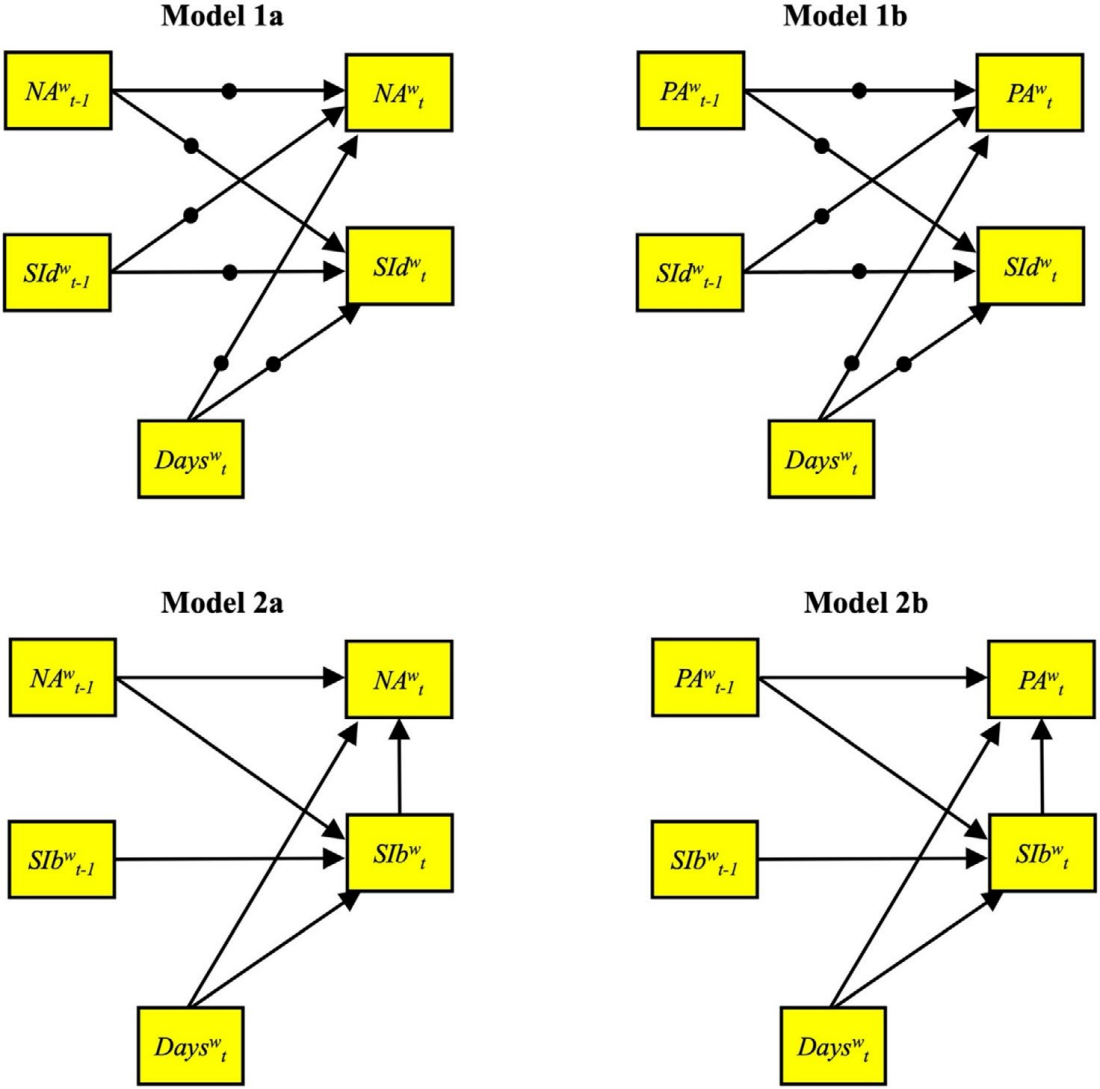
Path diagram for the reciprocal effects DSEM models (1a–2b). Only within‐person regression parameters are shown. NA = negative affect; PA = positive affect; SI = suicide ideation. Random effects are denoted by solid circles. Models 1a and 1b show reciprocal effects between NA/PA and dimensional SI severity (SId), which were all measured “at this moment”. Models 2a and 2b show reciprocal effects between NA/PA measured “at this moment” and binary SI (SIb) measured “since my last completed survey”; hence, *SIb* ^*w*^_*t*_ captures binary SI that reportedly occurred in the time between *NA* ^*w*^_*t‐1*_ and *NA* ^*w*^_*t*_.

Separate models were run for PA and NA, and for the different methods of measuring SI (dimensional and binary). Figure 1 displays path diagrams showing the within‐person regression parameters estimated in Models 1a–2b. For dimensional SI (Models 1a and 1b), we specified random autoregressive effects as well as random cross‐lagged effects between NA/PA and SI from time *t*‐1 to *t* at the within‐person level. As with dimensional SI models, bidirectional models for binary SI (Models 2a and 2b) also specified cross‐lagged and autoregressive effects between SI and PA/NA, but with two differences. First, because binary SI was assessed retrospectively on a different time scale, binary SI models were specified such that SI at time *t* (i.e., since the last assessment, or *t*‐1), after controlling for SI at *t*‐1 (autoregressive effect of SI), predicted change in NA/PA assessed at *t* (“at this moment”) after controlling for NA/PA at *t*‐1 (autoregressive effect of NA/PA). Second, to achieve model convergence for bidirectional models with the binary SI outcome, which are more computationally complex, it was necessary to model regression parameters as fixed effects.

**FIGURE 2:**
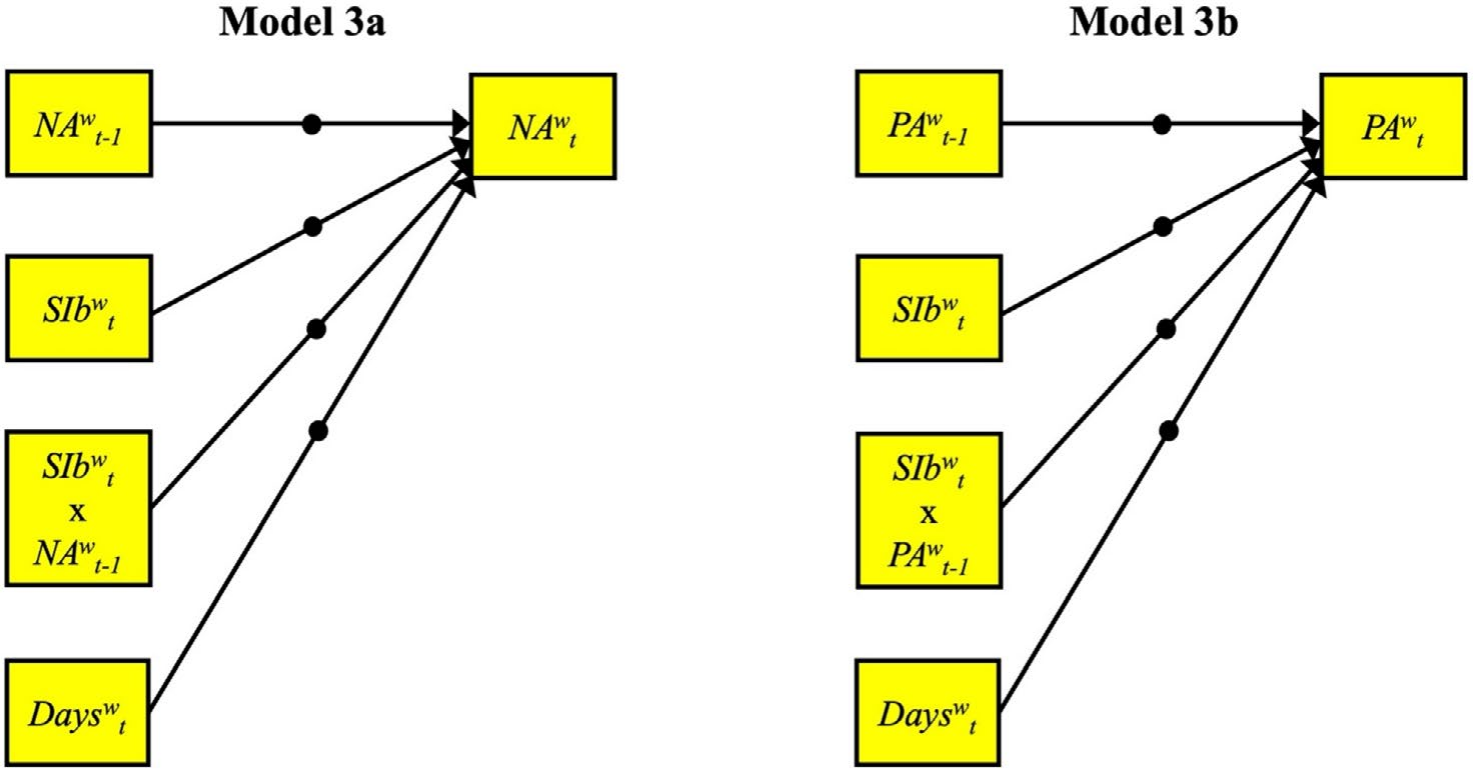
Path diagram for the moderation models (3a and 3b). Only within‐person regression parameters are shown. NA = negative affect; PA = positive affect; SI = suicide ideation. Random effects are denoted by solid circles. NA/PA were measured “at this moment” and binary SI (SIb) was measured “since my last completed survey”; hence, *SIb* ^*w*^_*t*_ captures binary SI that reportedly occurred in the time between *NA* ^*w*^_*t‐1*_ and *NA* ^*w*^_*t*_. The interactions (e.g., *SIb* ^*w*^_*t*_ × *NA* ^*w*^_*t‐1*_) capture moderation of the autoregressive effects for NA and PA by the occurrence of binary SI during each 2‐h time window.

Models 3a and 3b (see Figure 2 for path diagrams) examined whether experiencing SI during the *t*‐1 to *t* interval moderated the carryover effects of NA or PA (i.e., autoregressive effects of NA/PA_*t*‐1_ → NA/PA_*t*_), with all effects specified as random. In supplemental analyses, models with binary SI were replicated in the 81 participants who had endorsed this variable at some point during EMA to ensure that results were not biased by including those participants with no variance in binary SI.

Additional analyses were conducted to attempt replication of results from prior studies (e.g., Coppersmith et al. 2023; Kleiman et al. 2018; Kuehn, Dora, et al. 2022) that selected pairs of consecutive responses in which SI was endorsed and followed by no SI at the next timepoint, and examined whether affective experiences changed from during SI to after SI offset (i.e., differences in NA/PA during SI vs. after SI). We followed procedures of the previous studies as closely as possible considering differences between our EMA items measuring SI and items used in prior studies. For our purposes, to best identify presence versus absence of SI with the highest degree of reliability and validity, we defined the “during SI” moments as times when momentary “Desire to die by suicide right now” was rated nonzero and the binary active SI “since the last assessment” was also endorsed at the same timepoint. To be included in this analysis, the “during SI” responses had to be consecutively followed by no SI at the next time point, defined as not meeting the above‐defined criteria for an SI response. In addition, consistent with prior work, the consecutive pairs of assessments with SI followed by no SI had to be less than or equal to 4 h apart to be included in the analysis. There was a total of 223 response pairs (i.e., 446 total observations) across 56 participants that met these inclusion criteria. Following procedures described in the previous studies, we then examined NA and PA as dependent variables and event state (i.e., during or after SI) as a binary predictor in three‐level random effects mixed models with response pairs nested within events within participants. These models were estimated using IBM SPSS Statistics 29.

To identify potential covariates, we tested bivariate associations between demographic variables and EMA‐based dependent variables at the participant level. There were no significant associations (all *p*'s > 0.05) between mean levels of EMA variables (NA, PA, SI) and age, sex assigned at birth, gender identification, sexual orientation, race, ethnicity, receipt of public assistance tied to low income, or participation in EMA pre‐ versus post‐COVID‐19 pandemic. The correlation between mean EMA‐assessed SI and lifetime suicide attempt history approached significance (*r* = 0.16; *p* = 0.053); therefore, we included attempt history as a between‐persons covariate in dimensional SI DSEM models (regressing the latent mean for dimensional SI on attempt history, coded as 0 = *No attempt* and 1 = *One or more attempts*).

## 3 Results

Descriptive statistics and associations between person‐level study variables are shown in Table 3. Results from the primary DSEM models are shown in Tables 4 and 5.

**TABLE 3:** Descriptive statistics and bivariate correlations at the between‐person level (*N* = 140).

Variable	1	2	3	4	5	6
1. Attempt history	—					
2. Days	−0.11	—				
3. Negative affect	−0.03	−0.12	—			
4. Positive affect	−0.06	−0.08	−0.27**	—		
5. SI (dimensional)	0.16	−0.09	0.59**	−0.46**	—	
6. SI (binary)	0.08	0.23**	0.28**	−0.21*	0.41**	—
%/*M*	37.14%	9.78	34.80	39.73	17.62	0.04
SD		2.05	18.02	16.46	16.37	0.07
Range		1.32–21.41	5.86–82.62	1.17–85.65	0.02–69.94	0–0.52
Skewness		−0.56	0.47	0.09	0.80	3.62
Kurtosis		13.43	−0.33	0.13	−0.18	16.25

*Note:* Days = time elapsed since the first EMA assessment. Repeated measures from EMA were aggregated by person by calculating the mean across assessments. Mean for “SI binary” represents the proportion of observations where binary SI (coded 0 = no, 1 = yes) was endorsed, averaged across persons.

Abbreviation: SI = suicide ideation.

* *p* < 0.05.

** *p* < 0.01.

**TABLE 4:** Results of bidirectional effects models (*N* = 140).

Model	Predictor	Std est	SD	95% CI	% Pos	% Neg
1a	*DV = Dimensional SI at t*					
	Days	**−0.031**	0.008	−0.046, −0.015	10.7	17.9
	Dimensional SI at *t*‐1	**0.235**	0.011	0.213, 0.257	47.1	0.7
	Negative affect at *t*‐1	**0.143**	0.010	0.123, 0.162	31.4	1.4
	*DV = Negative affect at t*					
	Days	**−0.023**	0.008	−0.039, −0.008	8.6	16.4
	Dimensional SI at *t*‐1	**0.075**	0.013	0.049, 0.098	16.4	0.7
	Negative affect at *t*‐1	**0.413**	0.010	0.394, 0.432	96.4	0.0
1b	*DV = Dimensional SI at t*					
	Days	**−0.035**	0.008	−0.050, −0.019	10.0	20.0
	Dimensional SI at *t*‐1	**0.249**	0.011	0.228, 0.271	52.1	0.7
	Positive affect at *t*‐1	**−0.142**	0.010	−0.161, −0.123	0.0	32.1
	*DV = Positive affect at t*					
	Days	−0.005	0.008	−0.020, 0.011	11.4	15.0
	Dimensional SI at *t*‐1	**−0.057**	0.011	−0.079, −0.037	0.0	9.3
	Positive affect at *t*‐1	**0.398**	0.010	0.379, 0.418	86.4	0.0
2a	*DV = Binary SI at t (t‐1 to t)*					
	Days	**−0.019**	0.007	−0.032, −0.006		
	Binary SI at *t*‐1 (*t*‐2 to *t*‐1)	**0.856**	0.014	0.828, 0.884		
	Negative affect at *t*‐1	**0.050**	0.012	0.027, 0.074		
	*DV = Negative affect at t*					
	Days	0.007	0.011	−0.015, 0.029		
	Binary SI at *t* (*t*‐1 to *t*)	**0.405**	0.014	0.378, 0.431		
	Negative affect at *t*‐1	**0.336**	0.012	0.314, 0.360		
2b	*DV = Binary SI at t (t‐1 to t)*					
	Days	**−0.032**	0.008	−0.047, −0.017		
	Binary SI at *t*‐1 (*t*‐2 to *t*‐1)	**0.802**	0.018	0.766, 0.835		
	Positive affect at *t*‐1	**−0.084**	0.015	−0.116, −0.054		
	*DV = Positive affect at t*					
	Days	**−0.059**	0.011	−0.081, −0.037		
	Binary SI at *t* (*t*‐1 to *t*)	**−0.444**	0.016	−0.477, −0.412		
	Positive affect at *t*‐1	**0.263**	0.014	0.234, 0.289		

*Note:* Days = time elapsed since the first EMA assessment. All estimates are standardized. For models with random effects (Models 1a and 1b), estimates are within‐level standardized estimates averaged over clusters. Bolded estimates are statistically significant (i.e., 95% CIs do not cross zero). % Pos/Neg = of participants in the sample with significant positive or negative parameters (only available for random effects).

Abbreviations: CI = credibility interval; DV = dependent variable; NA = negative affect; PA = positive affect; SI = suicide ideation.

**TABLE 5:** Results of moderation models (*N* = 140).

Model	Predictor	Std est	SD	95% CI	% Pos	% Neg
3a	*DV = Negative affect at t*					
	Days	**−0.016**	0.007	−0.031, −0.002	7.9	13.6
	Binary SI at *t* (*t*‐1 to *t*)	**0.226**	0.016	0.193, 0.259	40.0	0.0
	Negative affect at *t*‐1	**0.445**	0.009	0.428, 0.460	98.6	0.0
	Interaction (SI*t* × NA*t*‐1)	−0.019	0.012	−0.042, 0.008	0.7	11.4
3b	*DV = Positive affect at t*					
	Days	−0.010	0.008	−0.026, 0.005	11.4	13.6
	Binary SI at *t* (*t*‐1 to *t*)	**−0.151**	0.013	−0.176, −0.126	0.0	51.4
	Positive affect at *t*‐1	**0.414**	0.009	0.396, 0.431	92.1	0.0
	Interaction (SI*t* × PA*t*‐1)	−0.016	0.012	−0.038, 0.007	0.0	5.0

*Note:* Days = time elapsed since the first EMA assessment. All estimates are within‐level standardized estimates averaged over clusters. Bolded estimates are statistically significant (i.e., 95% CIs do not cross zero). % Pos/Neg = of participants in the sample with significant positive or negative parameters.

Abbreviations: CI = credibility interval; DV = dependent variable; NA = negative affect; PA = positive affect; SI = suicide ideation.

### 3.1 Bidirectional Models (1a–2b; Table 4)

As hypothesized, we observed significant positive cross‐lagged associations between NA and subsequent dimensional SI severity (Model 1a) and binary SI likelihood (Model 2a), as well as negative cross‐lagged associations between PA and subsequent dimensional SI severity (Model 1b) and binary SI likelihood (Model 2b) within 2‐h intervals. In other words, within‐person elevations in NA and decreases in PA significantly predicted subsequent increases in SI severity and the likelihood of SI 2 h later. We also observed positive cross‐lagged associations between SI and subsequent changes in NA (Models 1a and 2a), and negative cross‐lagged associations between SI and subsequent changes in PA (Models 1b and 2b) within 2‐h intervals. In other words, as hypothesized, within‐person elevations in SI presence and severity were associated with subsequent *increases* in NA and *decreases* in PA 2 h later. However, as shown in Table 4, there was considerable heterogeneity in these effects, with most participants demonstrating no significant association between SI and subsequent change in affect. Because Models 2a and 2b were not estimated with random effects, individual heterogeneity is not available.

### 3.2 Moderation Models (3a and 3b; Table 5)

Results from moderation analyses demonstrated that experiencing SI during the *t*‐1 to *t* interval did not significantly moderate the carryover effects (i.e., autoregressive effects) of NA or PA from *t*‐1 to *t*. Contrary to an affect regulation hypothesis, these results suggest that experiencing active SI does not significantly influence the persistence of NA or PA over 2‐h intervals. Heterogeneity was again observed in these effects, but the majority had no significant interaction effect.

### 3.3 Supplemental Analyses

Supplemental analyses replicating Models 2a–3b in the subsample of 81 participants who had endorsed binary active SI at some point during EMA yielded the same substantive findings, suggesting that the above‐reported results were not biased by including those participants with no variance in binary SI.

### 3.4 Additional Analyses: Replication of Prior Findings

Additional analyses examining change in affect after instances of SI in pairs of consecutive assessments up to 4 h apart replicated findings from prior studies that found results consistent with an affect regulation model (Coppersmith et al. 2023; Kleiman et al. 2018; Kuehn, Dora, et al. 2022) As a reminder, this analysis included only pairs of consecutive responses where SI occurred at time *t* and was followed by no SI at *t* + 1. Consistent with prior work, results with this approach showed that NA decreased (*B* = −7.55, SE = 1.18, 95% CI = −9.87, −5.23; *p* < 0.001) and PA increased (*B* = 8.13, SE = 1.21, 95% CI = 5.74, −10.51; *p* < 0.001) after instances of SI (comparing “during SI” to “after SI”). Detailed results for all models are available at https://osf.io/7c4yq/.

## 4 Discussion

The existing literature examining an affect regulation model of SI is inconclusive, particularly regarding whether SI provides relief from affective distress. Prior studies have predominantly used one analytic approach that uses only selected observations of data constrained to instances when SI resolves from one timepoint to the next, and which does not account for autoregressive or bidirectional effects. To address the limitations of prior studies, we used a DSEM approach with EMA data from young adults with recent SI or suicidal behavior to examine reciprocal associations among SI and both NA and PA, as well as whether experiencing SI influenced the persistence of NA or PA over 2‐h intervals in daily life. As hypothesized, results demonstrated reciprocal effects between affect and SI. Increases in NA and decreases in PA were associated with significant increases in SI within 2 h, and SI was associated with *increases* in NA and *decreases* in PA 2 h later. These findings were consistent across distinct methods of assessing SI, whether SI was captured dimensionally across a full spectrum from passive to active suicidal thoughts or assessed using a single binary item capturing the retrospective occurrence of active SI in the 2‐h interval between consecutive assessments of affect. Further, in our exploratory moderation models, we found no evidence that experiencing active SI influenced the persistence of NA or PA over 2‐h intervals. These findings might be interpreted as running counter to an affect regulation model, as they do not support the idea that SI is reliably associated with short‐term decreases in NA (i.e., escape from aversive states) when considering all EMA observations over 2‐h intervals. Rather, our findings may suggest a maintenance model of SI, whereby affective distress and SI maintain each other over time via feedback loops or vicious cycles. However, the heterogeneity we observed in individual parameters also suggests that these processes operate differently for different people. In addition, there is variation in the duration and severity of SI, and the use of all available data for our DSEM models combines instances of prolonged SI with instances of fleeting SI, while accounting for changes or persistence in SI from one occasion to the next through autoregressive effects. Our results suggest that SI and affective distress are highly correlated and that the dynamic associations among these constructs are complex and highly variable within and between persons.

Our results provide a useful context and potential explanation for the inconsistencies noted in the existing literature, such that conclusions appear to greatly depend on the chosen analytic method, sampling strategy, and data inclusion choices. Importantly, when we analyzed our data using the approach used in prior studies (Coppersmith et al. 2023; Kleiman et al. 2018; Kuehn, Dora, et al. 2022) that constrained the data to only instances when SI resolves at the next timepoint (i.e., during SI at *t*‐1 followed by no SI at *t*), results matched those of the previous studies that would support an affect regulation model of SI by showing NA decreases and PA increases immediately following SI episodes. In other words, NA decreases and PA increases when individuals completely stop thinking about suicide. The limitation with this approach is that it fails to capture changes in affect across instances when SI emerges, gradually changes, or is maintained, as may naturally occur across time. It is also worth noting that such an approach greatly reduces the data being analyzed to only a small fraction of the intensive repeated measures. In our sample, this resulted in analyzing only 2.56% of total available EMA observations and reduced the sample size to 40% of people. This decrease in available data and sample size may reflect the relatively infrequent occurrence of this process. We also recognize that this approach has value because it differentiates these processes (i.e., fleeting SI vs. prolonged SI that continues over hours and days). It is a useful method for examining what happens when SI occurs and then ceases within a short period of time (i.e., 2–8 h).

However, with our DSEM models that used all available data rather than selected pairs of responses and explicitly modeled autoregressive and bidirectional effects, we reach very different conclusions. When we do this, our results are consistent with findings from some other studies suggesting that SI is closely followed by immediate increases in affective distress when using all available data (Al‐Dajani and Uliaszek 2021; Rath et al. 2019). Our DSEM results also replicate and extend findings by Coppersmith et al. (2023), who also found reciprocal effects between suicidal thinking and NA over 4‐h assessment intervals when accounting for autoregressive and reciprocal effects and using all available data. We extend these findings by showing that these reciprocal effects are observed in an independent sample over shorter intervals (2 h), for both NA and PA, and across two distinct ways of measuring SI.^1^ Our moderation analyses are another extension of prior work that has not been attempted in previous studies to our knowledge. The moderation models also incorporate all available data and autoregressive effects of NA and PA, and lead to similar conclusions to the bidirectional models, which run counter to an affect regulation model. These findings suggest that data inclusion/selection rules and whether models control for temporal and autoregressive effects are critically important design features that influence the interpretation of results.

Placing the current findings in the context of the existing literature, the most likely explanation of the findings across studies is that either an affect regulation (i.e., reinforcement) model or a maintenance model of SI may accurately account for the suicidal thinking process in different people depending on various individual characteristics, and/or at different times depending on various contextual circumstances. In fact, although our results based on average associations between momentary SI and affective experiences across individuals do not support an affect regulation model, we also observed considerable individual heterogeneity in these associations that may suggest SI functions to regulate affect for some individuals but not others. Recent studies using idiographic methods to build person‐specific models based on dynamic suicide risk factors suggest high levels of heterogeneity in short‐term suicide risk processes (Coppersmith et al. 2024; Kaurin et al. 2022b; Kuehn, Foster, et al. 2022; Kuehn et al. 2024; Yin et al. 2023). The small effect sizes observed in affect regulation models and discrepancies in results across studies may be indicative of such heterogeneity. Indeed, evidence suggests there are subgroups of individuals who report decreased distress, as well as those who report increased distress, following suicidal thinking (Crane et al. 2012, 2014; Hales et al. 2011), and there is also the possibility that many people experience both processes, just at different times. A possible interpretation of the mixed findings across studies and methods is that occasions when SI resolves and is followed by decreases in NA or increases in PA (i.e., affect regulation), even if relatively infrequent, maintain SI through powerful intermittent reinforcement. These occasions may be especially salient, even if relatively rare, and even if the more commonly observed dynamic is one where SI and affective distress are both more temporally persistent and highly correlated with each other across occasions. An important future direction is to examine this within‐ and between‐persons heterogeneity and its implications for predicting the longitudinal course of suicide risk using idiographic approaches such as Group Iterative Multiple Model Estimation (Gates and Molenaar 2012; Lane and Gates 2017), which could be used to identify subgroups with different dynamic associations between SI and NA/PA, and determine how those groups differ in the longitudinal course and patterns of recurrence of SI over time.

It is also possible that more severe SI such as intent or planning is more reinforcing than low‐level ideation. Our dimensional SI scale was comprised of two passive ideation items and one active ideation item, while several previous studies have incorporated more active ideation items including suicidal intent. However, this is unlikely to have influenced our results considering: (a) our results were consistent across dimensional SI and a binary measure of active SI; and (b) Coppersmith et al. (2023) found similar results for NA using DSEM with an SI score comprised of active SI and intent items. It is also possible that SI serves an affect regulatory function on a much shorter or much longer temporal scale. EMA studies using measurement burst or event contingent designs may be well suited to exploring whether SI may serve an affect regulation function on much shorter time scales, that is, over the course of minutes. Further, there are several other factors that would be important to directly test in future investigations of affect regulation and maintenance models of SI, including experiencing comfort from SI that may depend on the content of suicidal thinking (Crane et al. 2012, 2014), cognitions about SI, or the use or perceived effectiveness of SI as an affect regulation strategy based on the participant's own subjective report. The possibility that results differ depending on the type of NA, consistent with one prior study (Al‐Dajani and Uliaszek 2021), is also a useful future direction to pursue. We also note that recent EMA work has identified discrepancies between past retrospective reports of self‐reported functions of behavior and what is shown in modeling of real‐time EMA data, suggesting that there are significant challenges with translating functional models of clinical behaviors into statistical models of EMA data (Dora et al. 2023; Hepp et al. 2020). Thus, we caution against interpreting our results as a firm rejection of an affect regulation model of SI. Rather, we urge researchers to continue to examine both affect regulation and maintenance processes using a variety of methods and samples in the effort to further clarify the boundaries of effects demonstrated in this and prior studies and to explore heterogeneity of these processes.

There are several notable strengths of the current study that address limitations in prior work, including the relatively large sample size compared to prior studies testing an affect regulation model, greater density of EMA assessments (which allowed us to test effects across shorter time intervals compared to prior studies), use of all available data rather than selected pairs of responses, and explicit modeling of autoregressive and bidirectional effects using a powerful analytic approach. An additional strength is our measurement of SI using two distinct approaches, i.e., momentary dimensional and categorical, which yielded consistent findings across these methods. Further, we examined the temporal dynamics of SI in relation to changes in PA as well as NA, which has rarely been done in prior work but has important implications regarding desired affect change and potential treatment targets. Perhaps most importantly, we were able to show that divergent modeling approaches with the same dataset yield contradictory conclusions, and this will be important to consider in future research and theory building.

In terms of limitations, our participants were predominantly female and limited to adults 35 and younger. Thus, findings may not generalize to men, children or adolescents, or older adults. We also restricted our sample to those in active behavioral treatment, which enhances relevance to those seen in clinical practice, but limits the generalizability of our findings to all individuals in the general population with suicidal thoughts or behaviors. Future studies should attempt to replicate our results in samples that include more men and a wider age range, as well as those who are not receiving behavioral treatment. In addition, although our sample was high risk by virtue of having SI and/or behavior in the past 4 months (and included those at high acute risk), future studies should attempt to replicate our results in samples with more individuals with highly acute suicide risk (e.g., among samples with more recent suicide attempts, or among psychiatric inpatients). We also observed low base rates of active SI on the binary item during EMA, which is not uncommon, even in high‐risk samples such as ours. However, this did not appear to bias our results, which consistently showed significant effects in the opposite direction of what would be expected from an affect regulation model, and effects with the binary SI item were consistent with those from models with our dimensional SI measure.

These results may have important theoretical and clinical implications. Our findings suggest that SI may not be an objectively effective emotion regulation strategy for some people or in all circumstances, as it may not always provide relief from affective distress as expected based on the affect regulation model. However, there is heterogeneity in these processes, and there is a substantial need to tackle the translational gap between individualized models and clinical care (Piccirillo and Rodebaugh 2019). Monitoring fluctuations in SI and affect over time in treatment may be beneficial for identifying person‐specific patterns that could point to actionable treatment targets in personalized care. If such patterns point to feedback loops in a given individual, this data could be used to motivate change toward the use of alternative affect regulation strategies in patients by demonstrating that SI itself is not bringing about the desired escape from emotional suffering. A reciprocal effects pattern could also suggest that intervention could focus on reducing either SI or affective distress, as reducing one may influence change in the other. If an individual's data suggest that SI does reduce affective distress in the short term, treatment could focus on identifying and mitigating stressors and increasing distress tolerance skills. In the absence of other concerning risk factors (e.g., increases in SI intent or planning), clinicians may prioritize introduction of behavioral activation strategies to improve mood or alternative affect regulation strategies prior to directly reducing SI and its use as a coping mechanism. Removing SI that functions to reduce distress too quickly without replacement emotion regulation strategies may elevate distress. In either case, alternative affect regulation strategies can be explored, but the actual interventions may look different depending on whether reinforcement or feedback loops appear more relevant. The current findings suggest the need to carefully consider the limitations of distinct analytic methods when evaluating the affect regulation model and other prominent theories of suicide risk.

## Author Contributions

**Lori N. Scott:** conceptualization (lead), data curation (equal), formal analysis (lead), funding acquisition (lead), investigation (lead), methodology (lead), project administration (lead), writing – original draft (lead). **Sarah L. Brown:** conceptualization (supporting), data curation (equal), funding acquisition (supporting), investigation (supporting), methodology (supporting), project administration (supporting), writing – original draft (supporting).

## Ethics Statement

The University of Pittsburgh approved this study on November 30, 2018 and was assigned #STUDY18100158. All participants provided voluntary consent before participating in research activities.

## Conflicts of Interest

The authors declare no conflicts of interest.

## Data Availability

Detailed results and analysis code are publicly available on the Open Science Framework at https://osf.io/7c4yq/. Deidentified data used to produce these results are available directly from the corresponding author upon request.

## Endnotes

^1^
In sensitivity analyses, we explored results of our DSEM models using different time intervals from 1 to 8 h using the TINTERVAL command. Models with a 1‐h time interval did not converge, which was not surprising because this was too short of a window to adequately match our data. Results were very similar in magnitude and direction at 3‐ to 8‐h intervals to the 2‐h interval, with no difference in substantive interpretation across these intervals.
